# Endoscopic Bariatric Therapies for Metabolic Dysfunction-Associated Steatotic Liver Disease: Mechanistic Insights and Metabolic Implications

**DOI:** 10.3390/biomedicines13102437

**Published:** 2025-10-07

**Authors:** Wissam Ghusn, Mira Sridharan, Rachel Fromer, Muhammet Ozdemir, Madeleine G. Haff, Eric J. Vargas

**Affiliations:** 1 Department of Internal Medicine, Boston University, Boston, MA 02118, USA; wissamghusn7@gmail.com (W.G.); mira.sridharan@bmc.org (M.S.); rachel.fromer@bmc.org (R.F.); muhammet.ozdemir@bmc.org (M.O.); 2 Division of Gastroenterology and Hepatology, Mayo Clinic, Rochester, MN 55905, USA; 3 Division of Gastroenterology and Hepatology, Boston Medical Center, Boston, MA 02118, USA; madeleine.haff@bmc.org

**Keywords:** MASLD, endoscopic bariatric therapies, endoscopic sleeve gastroplasty, intragastric balloon, duodenal mucosal resurfacing, obesity

## Abstract

Metabolic dysfunction-associated steatotic liver disease (MASLD) is rapidly emerging as the leading cause of chronic liver disease, closely tied to rising global obesity rates. Endoscopic bariatric therapies (EBTs), including endoscopic sleeve gastroplasty (ESG), intragastric balloons (IGB), duodenal-jejunal bypass liners (DJBL), and duodenal mucosal resurfacing (DMR), offer minimally invasive interventions that target metabolic dysfunction and weight loss. This review synthesizes current evidence on the mechanisms and hepatic outcomes of EBTs in MASLD, highlighting improvements in hepatic steatosis, liver stiffness, and fibrosis biomarkers across multiple modalities. ESG is consistently associated with reductions in hepatic steatosis and fibrosis scores across multiple studies. IGB therapy improves liver stiffness and reduces hepatic fat as assessed by imaging modalities such as MRI- Proton Density Fat Fraction and ultrasound. DJBL lowers liver enzymes and improves non-invasive markers of steatohepatitis like the Fibroscan-AST score, although its effect on fibrosis appears limited. DMR demonstrates reductions in liver fat, particularly in patients with type 2 diabetes, but evidence for histological improvement in MASLD remains inconsistent. Despite their promise, most EBT studies remain limited by small sample sizes and short follow-up. Further randomized trials are needed to validate long-term efficacy and position EBTs alongside or as alternatives to surgical interventions for MASLD.

## 1. Introduction

The global prevalence of obesity continues to rise at an alarming rate, with significant implications on cardiovascular and metabolic health [1] and mortality [2]. Among the most prevalent hepatic manifestations of obesity is metabolic dysfunction-associated steatotic liver disease (MASLD), a condition that now affects nearly one in three individuals worldwide [3]. MASLD encompasses a broad clinical spectrum, from simple steatosis to metabolic dysfunction-associated steatohepatitis (MASH), progressive fibrosis, and cirrhosis, and is projected to become the leading indication for liver transplantation in the coming decades.

In parallel, the growing demand for effective yet less invasive weight loss solutions has led to the emergence of endoscopic bariatric therapies (EBTs). These interventions, which include procedures such as endoscopic sleeve gastroplasty (ESG), intragastric balloons (IGB), duodenal-jejunal bypass liner (DJBL) and duodenal mucosal resurfacing (DMR), offer a novel therapeutic option for patients with obesity who may not meet criteria for or prefer to avoid metabolic and bariatric surgery [4]. Their impact on weight reduction, glycemic control, and other metabolic parameters makes them particularly promising in addressing MASLD, which is intrinsically linked to systemic metabolic dysfunction [5].

Given the high burden and multisystemic nature of MASLD, this narrative review focuses on the rationale and evidence supporting the role of EBTs in its management. Understanding how these therapies modulate the underlying pathophysiology of MASLD may help clarify their place in the expanding treatment landscape for metabolic liver disease.

## 2. Pathophysiology of MASLD

MASLD represents a continuum of liver pathology ranging from simple hepatic steatosis to steatohepatitis, progressive fibrosis, and ultimately cirrhosis [6]. The transition from benign fat accumulation to inflammation and fibrosis is driven by a constellation of metabolic insults (e.g., insulin resistance, adipose tissue dysfunction, and chronic low-grade inflammation).

Insulin resistance plays a central role by promoting increased hepatic de novo lipogenesis, impaired lipid oxidation, and reduced export of triglycerides, all of which contribute to hepatic fat accumulation [7]. Concomitant dyslipidemia, characterized by elevated triglycerides and decreased high-density lipoprotein (HDL), further exacerbates lipid deposition in hepatocytes [8]. Lipotoxicity, oxidative stress, and mitochondrial dysfunction lead to hepatocyte injury, triggering inflammatory pathways and activation of hepatic stellate cells that promote fibrogenesis.

The gut-liver axis has also emerged as a critical component in MASLD pathogenesis [9]. Alterations in gut microbiota, intestinal permeability, and microbial-derived metabolites may contribute to hepatic inflammation and fibrosis through increased translocation of endotoxins and activation of innate immune responses [10]. This underscores the systemic nature of MASLD and provides a mechanistic link between obesity, metabolic syndrome, and progressive liver injury [1].

Weight loss plays a pivotal role in the management of MASLD by directly addressing the underlying pathophysiology of hepatic fat accumulation and insulin resistance. Even modest reductions in body weight, on the order of 5–10 percent of total body weight, have been shown to significantly decrease hepatic steatosis, improve liver enzyme profiles, and attenuate inflammatory pathways that drive disease progression [11]. As adipose tissue shrinks, the influx of free fatty acids into the liver diminishes, reducing triglyceride deposition and alleviating lipotoxic stress on hepatocytes [12]. Furthermore, weight loss enhances peripheral insulin sensitivity, thereby disrupting the vicious cycle in which hepatic insulin resistance contributes to ongoing fat synthesis and inflammation [13]. Collectively, sustained weight reduction not only mitigates early-stage steatosis but also promotes regression of fibrosis, underscoring its essential role in preventing long-term complications such as cirrhosis and hepatocellular carcinoma.

## 3. Mechanistic Basis of Weight Loss-Induced MASLD Improvement

### 3.1. Caloric Restriction and Fat Mobilization

Calorie restriction, most commonly implemented via a hypocaloric diet that reduces daily energy intake by 500–1000 kcal or targets an absolute intake of 1200–1500 kcal/day, consistently reduces hepatic steatosis, enhances insulin sensitivity, and lowers transaminase levels in patients with MASLD [14]. As a fundamental component of MASLD management, structured weight-loss programs emphasize four core objectives: (1) accurate baseline weight assessment, (2) intensive caloric reduction to achieve ≥5–10% total body weight loss (TBWL), (3) weight stabilization once target weight is reached, and (4) prevention of weight recurrence [15]. Randomized controlled trials demonstrate a clear dose–response relationship between weight loss and liver histology: ≥5% TBWL correlates with measurable improvement in steatosis; ≥7% TBWL often corresponds to resolution of MASLD; and ≥10% TBWL is linked to regression of fibrosis [16,17,18,19,20]. In younger patients, dietary quality changes, beyond sheer caloric reduction, show the strongest association with fibrosis improvement.

Mechanistically, caloric restriction mitigates MASLD by interrupting the pathologic cycle of unrestrained lipolysis and hepatic triglyceride accumulation. Under insulin-resistant conditions, excessive adipose lipolysis releases free fatty acids (FFAs) to the liver, where heightened re-esterification exacerbates intracellular triglyceride deposition, steatosis, and fibrogenesis [21,22]. Conversely, caloric restriction improves insulin sensitivity, measured by Homeostatic Model Assessment for Insulin Resistance (HOMA-IR) or clamp techniques, in both cohorts with and without obesity, thereby reducing FFA flux to hepatocytes and enabling partial reversal of lipid overload [23,24].

However, maintaining continuous caloric restriction poses significant adherence challenges. Intermittent energy restriction regimens can yield comparable improvements in hepatic fibrosis to continuous restriction, though without markedly better compliance [16]. For patients with comorbidities or physical limitations that preclude strict dietary interventions, professional guidelines from the American Gastrointestinal Association (AGA) and American Association for the Study of Liver Disease (AASLD) recommend early consideration of pharmacologic or procedural therapies to achieve and maintain adequate weight reduction [14].

### 3.2. Hormonal Modulation

Although insulin resistance remains a hallmark of MASLD pathophysiology, its interplay with hormonal networks is multifaceted and continues to unfold. Emerging evidence highlights the significant roles of various endocrine axes, including sex hormones, thyroid hormones, and others, in both the development and progression of MASLD. Below, we summarize key hormonal interactions that have been most extensively studied in relation to MASLD.

### 3.3. Ghrelin

**Figure 1:**
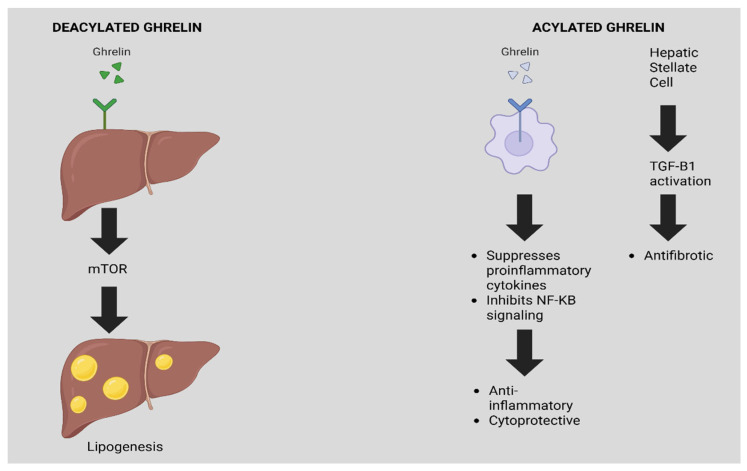
Steatogenic and antifibrotic pathways of de-acylated versus acylated ghrelin in MASLD.

Ghrelin, a gastric-derived hormone, exerts a complex and context-dependent influence on MASLD. Its metabolic effects are largely dictated by its molecular form, acylated versus deacylated. Notably, the deacylated form has been implicated in promoting hepatic lipogenesis via the mTOR signaling pathway, thereby contributing to steatosis in both animal and human studies. Consistently, murine models with ghrelin gene silencing exhibit marked reductions in hepatic fat accumulation, reinforcing its pathophysiologic role in MASLD (Figure 1).

In contrast, ghrelin also exerts anti-inflammatory and cytoprotective effects. It suppresses the production of pro-inflammatory cytokines, inhibits NF-κB signaling, and reduces hepatic apoptosis and autophagy. In hepatic stellate cells, acylated ghrelin attenuates TGF-β1-mediated activation and fibrogenesis, thus offering a potential antifibrotic mechanism. However, despite these protective actions, ghrelin’s upstream role in promoting steatosis may predominate in the setting of chronic metabolic stress. Indeed, elevated circulating ghrelin levels have been associated with more severe MASLD phenotypes, suggesting that its beneficial effects may be context-dependent and ultimately insufficient to counteract disease progression.

### 3.4. Fibroblast Growth Factor 19 and 21 (FGF19/FGF21)

FGF19 is secreted postprandially from enterocytes in response to bile acid signaling and works to suppress bile acid synthesis, inhibit hepatic lipogenesis, and improve insulin sensitivity. Reduced FGF19 levels are correlated with worsening MASLD [25]. In line with that, circulating serum FGF19 concentrations were lower in patients with obesity independent of their degree of insulin resistance [26]. FGF19 analogs as a treatment modality for MASLD are still under investigation.

**Figure 2:**
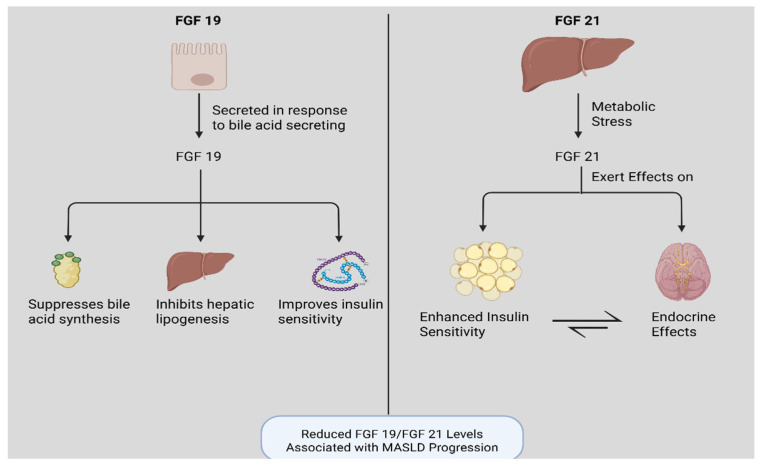
FGF19 and FGF21 metabolic pathways and their link to MASLD.

Moreover, FGF21 is a hepatokine secreted in response to metabolic stress, particularly in the presence of elevated free fatty acids [27]. It enhances insulin sensitivity and modulates lipid metabolism, thereby mitigating hepatic injury [28]. Uniquely, FGF21 exerts endocrine effects on both adipose tissue and the central nervous system [29]. Clinically, circulating FGF21 levels correlate with histologic severity in MASLD and may serve as a biomarker for fibrosis progression; higher levels are associated with greater hepatic injury, and certain genetic variants may predispose to early damage [27]. FGF21 levels also inversely correlate with weight changes; greater reductions in weight are associated with steeper declines in FGF21 [30]. Importantly, FGF21 analogs are under investigation as therapeutic agents in MASLD, with early-phase trials showing encouraging metabolic and histologic improvements (Figure 2) [31].

### 3.5. Glucagon-like Peptide 1 (GLP-1)

**Figure 3:**
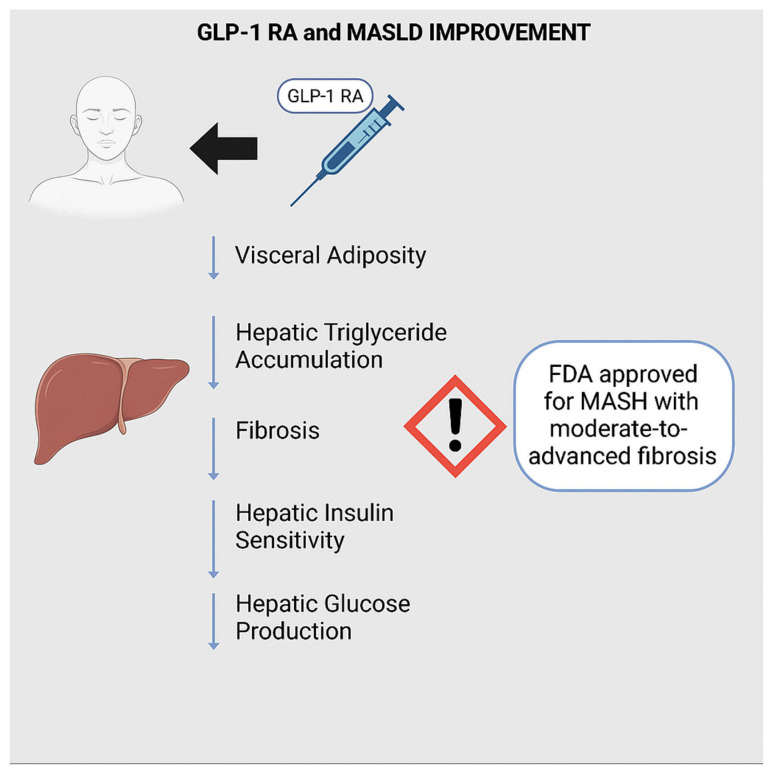
GLP-1 RA and MASLD Improvement.

GLP-1 is a hormone produced in enteroendocrine L cells in the ileum and colon with a role in promoting insulin secretion and satiety [32]. Although hepatocytes do not directly express GLP-1 receptors, there is overwhelming evidence to suggest that the use of GLP-1 receptor agonists (GLP-1RA; e.g., semaglutide) is correlated with improved hepatic steatosis and slowing of fibrosis [33,34,35,36,37]. GLP-1RA likely improve MASLD indirectly by suppressing appetite, reducing visceral adiposity, and enhancing insulin sensitivity, mechanisms that collectively decrease hepatic triglyceride accumulation [38]. Additionally, GLP-1 RAs have been shown to modulate the gut microbiome, which may further contribute to MASLD regression, although this pathway remains under active investigation [34]. Importantly, GLP-1 RAs have been recently FDA-approved MASH, particularly for patients with moderate to severe fibrosis (Figure 3) [39].

### 3.6. Peptide YY (PYY)

PYY secreted by intestinal L-cells, plays a key role in lipid homeostasis and energy balance. PYY exists in two principal isoforms: PYY (1-36) and PYY (3-36). The latter, PYY (3-36), potently suppresses appetite and reduces caloric intake. Disruption of normal PYY processing, resulting in reduced PYY (3-36) activity, is associated with increased adiposity and insulin resistance, which can exacerbate hepatic lipid accumulation and promote MASLD progression.

**Figure 4:**
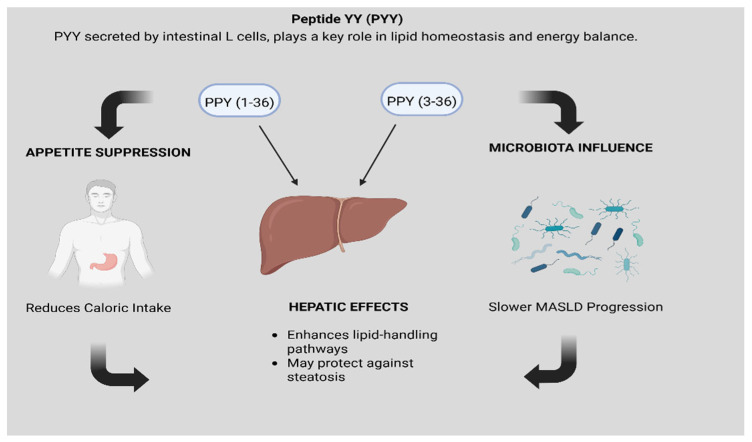
PYY’s effect on lipid homeostasis and energy balance.

PYY also exerts direct hepatic effects by enhancing lipid-handling pathways. It appears to upregulate key lipogenic enzymes, such as acetyl-CoA carboxylase, while modulating AMP-activated protein kinase activity, which together facilitate more efficient triglyceride processing and may protect against steatosis [40]. Additionally, PYY influences gut microbiota composition; emerging evidence links these microbiome shifts to slower MASLD progression, suggesting that PYY’s benefits extend beyond appetite suppression to include direct metabolic and microbial mechanisms (Figure 4).

### 3.7. Microbiome

Gut dysbiosis, small intestinal bacterial overgrowth, and shifts in microbial metabolites are consistently linked to MASLD progression, although the exact mechanisms remain unclear [41]. In particular, patients with worsening MASLD tend to have lower overall microbial diversity, with overrepresentation of genera such as *Escherichia*, *Prevotella*, *Lachnoclostridium*, and *Dorea* and underrepresentation of *Akkermansia muciniphila* and *Faecalibacterium* [10,41,42]. These compositional changes likely disrupt bile acid signaling and short-chain fatty acid (SCFA) production. Reduced SCFA levels, for instance, promote hepatic inflammation [10,41,43]. Intriguingly, Dong et al. found that, although sustained weight loss modestly altered gut flora, certain taxa (e.g., *Escherichia/Shigella*, *Klebsiella*, *Megasphaera*, and *Actinomyces*) persisted disproportionately in patients who failed to lose significant weight or improve steatosis [44]. This suggests that specific microbial signatures may identify individuals at risk for treatment-resistant MASLD and guide targeted interventions.

Surgical weight-loss interventions also remodel the gut microbiome in ways that favor metabolic health. After bariatric procedures, populations of *Akkermansia muciniphila* and *Bifidobacterium species*, both of which support intestinal barrier integrity and enhance insulin sensitivity, tend to flourish [45]. These shifts boost SCFA production, dampening systemic inflammation [46]. Additionally, surgery-induced changes in bile-acid circulation further modulate gut, endocrine signaling, promoting the release of anti-inflammatory hormones such as GLP-1.

### 3.8. Endoscopic Bariatric Therapies (EBTs)

EBTs encompass a range of minimally invasive, endoscopic procedures designed to help patients with obesity achieve sustained weight loss and metabolic improvement without the need for traditional surgery. By targeting the gastrointestinal tract, either by reducing stomach volume (e.g., IGB, ESG), altering nutrient flow (e.g., DJBL), or modifying mucosal signaling (e.g., DMR), EBTs promote earlier satiety, slower gastric emptying, and enhanced insulin sensitivity (Table 1) [47]. Importantly, EBTs typically carry lower perioperative risk, shorter recovery times, and the potential for reversibility or repeat intervention compared to surgical alternatives [47].

### 3.9. Endoscopic Sleeve Gastroplasty (ESG)

Endoscopic sleeve gastroplasty (ESG) employs an endoscopic suturing device to create a tubular, reduced-calorie reservoir by plicating the greater curvature of the stomach. Unlike surgical sleeve gastrectomy, ESG preserves the anatomy of the stomach without any incisions or gastric resection, leading to fewer perioperative risks and faster recovery. By decreasing gastric volume and altering gastric motility, ESG induces earlier satiety and prolongs the time to gastric emptying, which can translate to consistent weight loss of 15–20 percent total body weight over 12–18 months in many patients.

### 3.10. Intragastric Balloon (IGB)

The intragastric balloon (IGB) therapy involves the placement of a saline- or gas-filled silicone balloon into the stomach, which remains in place for approximately six months. The balloon occupies space within the gastric lumen, decreasing the available volume for food and triggering stretch receptors that signal fullness to the brain, promoting reduced caloric intake. Clinically, IGBs typically yield an average of 10–15 percent TBWL during the indwelling period, with some designs allowing for adjustment of balloon volume to tailor tolerability and efficacy [48].

### 3.11. Duodenal-Jejunal Bypass Liner (DJBL)

The duodenal-jejunal bypass liner (DJBL) is a 60 cm impermeable sleeve that is endoscopically anchored in the duodenal bulb and extends into the proximal jejunum. By creating a physical barrier between chyme and the duodenal-jejunal mucosa, DJBL bypasses the proximal small intestine for nutrient exposure, thereby replicating aspects of the Roux-en-Y gastric bypass without surgical anastomoses or gastric pouch formation. This results in early improvements in glycemic control by altering entero-insular signaling, reducing inappropriate insulin secretion, and enhancing peripheral insulin sensitivity. Over a 12-month indwelling period, many patients achieve 20–25 percent reduction in excess body weight [47], or 12.6% of TBWL [49], along with significant decreases in hemoglobin A1c, triglycerides, and hepatic steatosis [47].

### 3.12. Duodenal Mucosal Resurfacing (DMR)

Duodenal mucosal resurfacing (DMR) uses a specialized catheter to deliver controlled hydrothermal ablation to a segment of the duodenal mucosa, thereby stripping and regenerating the epithelial lining. This selective mucosal ablation disrupts maladaptive nutrient sensing and neurohormonal signals that contribute to insulin resistance, independent of major alterations in gastric volume or food absorption. Clinical studies of DMR have demonstrated early improvements in glycemic indices, average hemoglobin A1c reductions of around 1%, as well as modest decreases in liver enzymes and hepatic fat content in patients with metabolic dysfunction. Over 6–12 months, many patients can expect around 5% TBWL, partly secondary to improved glucose homeostasis and satiety signaling [47].

## 4. EBTs and MASLD: Emerging Evidence

### 4.1. Mechanistic Pathways for Improvement in MASLD Outcomes

Bariatric surgery induces weight loss through more than simple calorie restriction. In the early postoperative period, it triggers rapid changes in gut hormone profiles, intestinal microbiota composition, and bile acid metabolism, all of which work together to regulate appetite, enhance satiety, restore gut homeostasis, and alter lipid absorption [50,51]. EBTs aim to replicate these metabolic and physiological effects in a less invasive manner, drawing directly on the mechanisms uncovered by their surgical predecessors.

EBT trials have consistently demonstrated improvements in weight loss, insulin sensitivity, and fibrosis, yet the underlying physiologic mechanisms by which EBT affects MASLD outside of caloric-restriction based weight loss remain poorly understood. EBT includes both space-occupying techniques (e.g., IGB, ESG) that promote caloric restriction or small-bowel restructuring methods (e.g., DMR, DJBL) that directly alter nutrient absorption and affect hormonal changes [50]. However, there is a striking paucity of studies delineating the specific mechanisms at play, particularly in terms of long-term outcomes. Current research efforts aim to correlate shifts in gut microbiota with immunologic changes that might underlie MASLD improvement, while other groups have performed small-intestinal biopsies to determine whether EBT-induced intestinal remodeling influences duodenal mucosal differentiation and lipogenic signaling pathways [52,53]. Ultimately, the goal of this work is to enable clinicians to tailor EBT interventions to each patient’s unique pathogenic drivers of MASLD progression.

### 4.2. Current EBT Outcomes in MASLD

While numerous studies have evaluated the impact of surgical bariatric procedures on MASLD, far fewer have examined liver-specific outcomes following EBTs. Existing EBT studies often involve small cohorts and focus on individual techniques, most commonly ESG, yet they nonetheless suggest promising improvements in MASLD-related biomarkers [14,54]. Many of the studies were conducted before the adoption of MASLD, and include the old terminology of non-alcohol associated fatty liver disease (NAFLD), and are referenced as such. In a meta-analysis involving patients with obesity undergoing FDA-approved EBTs (i.e., IGB, ESG, and aspiration therapy), liver fibrosis was significantly reduced (mean difference 0.7; 95% CI, 0.1–1.3; *p* = 0.02). Secondary outcomes included a mean ALT decrease of 9.0 U/L (95% CI, –11.6 to –6.4; *p* < 0.0001), a reduction in hepatic steatosis (mean difference 1.0; 95% CI, –1.2 to –0.8; *p* < 0.0001), and a decrease in NAFLD activity score by 2.5 points (95% CI, –3.5 to –1.5; *p* < 0.0001), with accompanying improvements in insulin resistance and waist circumference.

Patient-level variability in outcomes after EBTs is increasingly recognized. Factors such as genetic background, baseline fibrosis stage, insulin resistance phenotype, and gut microbiome composition may determine the degree of weight loss and hepatic improvement achieved [55]. For instance, microbial signatures persisting after weight loss interventions have been associated with treatment resistance. Future work integrating genomic and microbiome profiling may enable precision-medicine approaches to select patients most likely to benefit.

### 4.3. ESG

In a 40-patient randomized trial, Abad et al. reported that ESG combined with a lifestyle intervention significantly reduced liver stiffness and histological steatosis versus sham endoscopy plus lifestyle modification. Notably, the Non-Alcoholic Steatohepatitis Activity (NAS) score only improved in the ESG group when controlling for weight loss exceeding 10% [14]. Similarly, Hajifathalian et al. prospectively followed 118 patients with MASLD for two years after ESG [56]. Both the Hepatic Steatosis Index and NAFLD Fibrosis Score declined significantly over that period, by roughly 4 and 0.3 points per year, respectively, and over 20% of participants regressed from fibrosis stages F3–F4 to F0–F2 [56]. In a systematic review and meta-analysis, among 175 patients with obesity and MASLD treated with ESG, 12-month follow-up showed significant reductions in hepatic steatosis index (−4.85; 95% CI −6.02 to −3.67), NAFLD fibrosis score (−0.50; 95% CI −0.80 to −0.19), and ALT (−6.32 U/L; 95% CI −9.52 to −3.11). ESG also produced 17% TBWL, 6.3 kg/m^2^ body mass index (BMI) reduction, 48% excess weight loss, and a 0.5% decrease in Hemoglobin A1c (HbA1c) (all *p* < 0.05) [57].

In 26 adults with obesity and MASLD undergoing ESG, mean ALT decreased from 59.5 IU/L at baseline to 49.5 IU/L at 6 months and 48.4 IU/L at 12 months (*p* = 0.001). Mean nonalcoholic fatty liver disease fibrosis score (NFS) improved from 0.228 to –0.202 and –0.552 at 6 and 12 months, respectively (*p* = 0.001); mean hepatic steatosis index (HSI), fibrosis-4 index (FIB-4), and aspartate aminotransferase-to-platelet ratio index (APRI) similarly decreased from baseline to 6 and 12 months (all *p* = 0.001). Patients achieved 18.1% total body weight loss at 12 months and showed improved HbA1c, with no major adverse events reported [58]. In a retrospective analysis of patients with MASLD and established fibrosis, Jirapinyo et al. found that ESG combined with GLP-1RA led to significant improvements in imaging-based fibrosis assessments (i.e., transient elastography) and reductions in serum transaminases (e.g., AST, ALT) at 6–12 months post-procedure [59]. Similarly, other studies on ESG with obesity pharmacotherapy have shown promising weight loss outcomes.

### 4.4. IGB

In patients with obesity and MASLD with advanced fibrosis, 6 months of fluid-filled intragastric balloon therapy led to reduced body weight from 106 ± 19.7 kg to 92 ± 18.3 kg (−14 kg, *p* < 0.001) and waist circumference from 116 ± 13.3 cm to 104 ± 13.4 cm (−12 cm, *p* < 0.001). Fibrosis markers improved: liver stiffness decreased from 13.3 ± 3.2 kPa to 11.3 ± 2.8 kPa (−2 kPa, *p* < 0.001) and FIB-4 from 3.2 ± 0.7 to 2.7 ± 0.8 (−0.5, *p* < 0.001) [60]. In a meta-analysis of 19 studies involving 911 patients, IGB therapy reduced NAFLD activity score by a mean of 3.0 points (95% CI: –2.41 to –3.59), ALT by 10.4 U/L (95% CI: –7.31 to –13.49), liver volume by 397.9 mL (95% CI: –212.78 to –1008.58), and liver steatosis by 37.8 dB/m (95% CI: –21.59 to –53.92). Significant non–liver-related improvements included decreases in body weight, BMI, HbA1c and HOMA-IR [61]. In another study, patients with obesity and histologically confirmed NASH were randomized to a cohort with a placement of Bioenterics Intragastric Balloon (BIB) versus identical diet/exercise with a sham procedure for six months. The BIB group experienced significantly greater BMI reduction (mean decrease in BMI: 1.52 vs. 0.8; *p* = 0.0008) and lower NAFLD activity scores at study end (median 2 vs. 4; *p* = 0.03), though no differences were seen in inflammation, ballooning, or fibrosis, indicating that larger, longer trials are needed [62]. In another prospective study, patients with obesity and MASLD (≥F2 fibrosis and/or S3 steatosis on transient elastography) were offered six months of lifestyle modification with or without an IGB based on preference, and 29 of 50 eligible patients underwent follow-up transient elastography. After six months, liver stiffness significantly decreased in the IGB group, from 6.0 kPa to 4.9 kPa (*p* = 0.005), but not in the lifestyle-only group, which had no significant change (5.5 kPa to 6.9 kPa; *p* = 0.477). Steatosis improved in both groups, with controlled attenuation parameter values decreasing from 328 ± 34 dB/m to 272 ± 62 dB/m in the IGB group and from 344 ± 33 dB/m to 305 ± 43 dB/m in the lifestyle-only group [63]. A meta-analysis across ten studies (nine observational and one randomized trial), IGB therapy in patients with obesity led to significant reductions in liver enzymes (ALT decreased by 10.02 U/L [95% CI: −13.2, −6.8]; GGT decreased by 9.82 U/L [95% CI: −12.9, −6.8]) and BMI (−4.98 kg/m^2^ [95% CI: −5.6, −4.4]) at six months. Imaging and histologic markers of MASLD also improved, with MRI-measured hepatic fat fraction falling from 16.7 ± 10.9% to 7.6 ± 9.8% (*p* = 0.003), severe steatosis on ultrasound dropping from 52% to 4% (*p* < 0.0001), and NAFLD activity score decreasing to 2 ± 0.75 versus 4 ± 2.25 in controls (*p* = 0.03) [64]. Interestingly, among 56 patients with compensated MASH cirrhosis, Spatz3™ IGB placement achieved a mean weight loss of 15.88 kg (−16.5%; BMI reduction −10.1%) at 6 months, with ≥10% TBWL in 55.4% of patients. Mean hepatic venous pressure gradient fell by 11.1% (14.2 ± 2.1 to 12.6 ± 1.7 mmHg), liver stiffness by 28.6%, and CAP by 10.1% [65]. Similarly, other studies have also shown significant improvement in weight loss outcomes and liver fibrosis in patients with MASLD.

### 4.5. DJBL

In one study, DJBL placement in patients with obesity and type 2 diabetes led to early and sustained improvements in MASLD-related plasma markers: at 3 months, AST fell by 7 IU/L, ALT by 22 IU/L, and γ-GT by 22 IU/L (all *p* < 0.05). Three months after device removal, ALT remained 17 IU/L below baseline, and γ-GT 24 IU/L below [66]. In another study, 32 patients were treated with DJBL for 48 weeks; AST decreased to 0.74 times baseline and ALT to 0.63 times baseline (both *p* < 0.001), and the Fibroscan-AST score fell by 0.21 (*p* < 0.001), indicating reduced steatosis and MASH activity. Fibrosis measures (e.g., LSM, NFS, ELF) remained unchanged, though FIB-4 showed slight improvement [67]. Further studies are needed to demonstrate the effect of these procedures on patients with MASLD.

### 4.6. DMR

In the multinational REVITA-2 trial, DMR led to a greater reduction in liver fat among patients with baseline MRI-PDFF > 5%. At 12 weeks, liver fat decreased by 5.4% in the DMR group versus 2.9% in the sham group; however, this difference was not statistically significant overall (*p* = 0.096). In a post hoc analysis, a significant reduction was observed in the European subgroup (*p* = 0.035), though this finding should be considered exploratory. These findings suggest that DMR may significantly reduce hepatic steatosis in patients with type 2 diabetes, and supports its potential role as a metabolic intervention targeting liver fat [68]. In another pilot study of 14 patients with biopsy-proven MASH, DMR without lifestyle intervention did not lead to MASH resolution at 12 months. A total 27% showed marginal improvement in fibrosis without worsening of MASH, with no significant changes in NAS score, liver enzymes, or metabolic markers [69]. In a meta-analysis of two studies including 67 participants, DMR demonstrated a non-significant reduction in liver fat with a mean change in MRI- Proton Density Fat Fraction of –2.22% (95% CI: –12.79 to 8.34), HbA1c by –0.32% (95% CI: –0.80 to 0.16), and HOMA-IR by 0.15 (95% CI: –5.11 to 5.41). While these findings suggest a potential metabolic benefit in patients with MASLD/MASH, the evidence remains limited and of very low quality.

## 5. Patient Selection for Endoscopic Bariatric Therapies

Patient selection is critical in determining the optimal EBT for individuals with MASLD. Restrictive procedures such as ESG are generally best suited for patients with moderate-to-severe obesity (BMI ≥ 30–35 kg/m^2^) who have not achieved durable weight loss with lifestyle interventions or pharmacotherapy. ESG may be particularly appropriate for patients with higher baseline fibrosis stages, where greater and sustained weight loss is needed to drive histological improvement.

In contrast, DMR, which primarily modifies mucosal signaling and glucose homeostasis, may be more appropriate for patients with type 2 diabetes, insulin resistance, or metabolic dysfunction, even in the absence of advanced obesity. Since weight loss from DMR is modest, its utility lies more in metabolic and glycemic control rather than in significant reductions in body weight or fibrosis regression.

IGB may be preferable for patients seeking a temporary, reversible option, particularly those with lower BMI ranges or those needing short-term weight loss to qualify for surgery or transplantation. DJBL may be suited for patients with severe insulin resistance and poor glycemic control, but their role in advanced fibrosis remains less clear.

Finally, patient preference and risk profile should guide decision-making. Patients averse to permanent anatomical alteration may favor reversible or temporary options, while those at higher procedural risk may benefit from minimally invasive, lower-risk EBTs. Ultimately, shared decision-making that considers BMI, fibrosis stage, metabolic comorbidities, and patient expectations is essential for selecting the most appropriate therapy.

## 6. Safety Considerations of Endoscopic Bariatric Therapies

While EBTs hold promise for MASLD management, their adoption must be weighed against procedure-specific safety profiles.

- ESG: ESG is generally well tolerated, with most adverse events limited to transient abdominal pain, nausea, or reflux symptoms. The need for hospitalization is low compared to surgical sleeve gastrectomy, but careful patient selection and post-procedure monitoring remain essential [70].
- IGB: Intolerance, manifested as persistent nausea, vomiting, or abdominal pain, is the most common reason for early removal. Balloon deflation with migration into the small bowel is rare but may lead to obstruction. Ulceration and, rarely, gastric perforation have also been described [48].
- DJBL: Although DJBL can significantly improve glycemic control and steatosis, adverse events remain a limiting factor. Device migration and spontaneous early removal occur in a small percentage of cases. Hepatic abscess formation, though uncommon, is one of the most serious complications and has led to early termination of some clinical trials. Other risks include GI bleeding and obstruction at the liner’s distal tip [71].
- DMR: DMR is generally well tolerated, with mild GI symptoms (e.g., abdominal pain, diarrhea) as the most frequent adverse events. However, mucosal injury carries a theoretical risk of strictures, ulceration, or delayed bleeding, underscoring the need for ongoing safety monitoring as this technology evolves [68].

Overall, while these therapies provide less invasive alternatives to surgery, their safety profiles must be considered alongside efficacy when selecting the most appropriate therapy for patients with MASLD.

## 7. Limitations

This review highlights the emerging role of endoscopic bariatric and metabolic therapies, but it is important to acknowledge the limitations of the existing evidence. Many studies in this field are small, single-center, or industry-sponsored, raising concerns regarding generalizability and potential bias. Furthermore, considerable heterogeneity exists across study designs, follow-up duration, and outcome reporting, with the majority relying on imaging- or biochemical-based surrogate markers rather than histological endpoints. Given these factors, the overall quality of evidence remains variable, and definitive conclusions regarding long-term efficacy and safety are limited. Large-scale, multi-center randomized controlled trials with standardized histological outcomes are needed to better establish the durability and clinical impact of these interventions.

In addition, the majority of EBT trials to date have focused on surrogate endpoints, such as improvements in liver enzymes, imaging-based steatosis, or non-invasive fibrosis scores, rather than hard outcomes like cirrhosis progression, hepatic decompensation, or liver-related mortality. In contrast, evidence from bariatric surgery cohorts has demonstrated reductions in long-term mortality in patients with MASLD and cirrhosis [72]. Whether EBTs can achieve similar disease-modifying benefits remains uncertain, underscoring the need for longer-term, adequately powered studies.

## 8. Conclusions

EBTs represent a promising frontier in the management of MASLD, offering minimally invasive, reversible alternatives to surgery for patients with obesity and metabolic dysfunction. These interventions consistently demonstrate meaningful reductions in hepatic steatosis, liver enzymes, and surrogate fibrosis markers, particularly with ESG and IGB, while DMR and DJBL show potential benefits in glycemic and metabolic control. However, current evidence remains limited by small sample sizes, heterogeneity of study design, and lack of long-term histologic outcomes. Large, well-controlled trials are essential to better define their efficacy and safety.

Looking ahead, the therapeutic landscape is rapidly evolving with the FDA approval of pharmacologic agents for MASH and the profound weight loss achieved with dual incretin agonists such as tirzepatide. In this context, the future role of EBTs is likely to be complementary rather than competitive with pharmacotherapy. Their greatest promise may lie in combination with GLP-1 receptor agonists or dual agonists, where additive or synergistic effects on weight loss, metabolic improvement, and liver outcomes could be realized. EBTs may serve as a platform to enhance pharmacotherapy response, a strategy to maintain weight loss after discontinuation of medications, or a rescue option for patients who experience partial response or intolerance to drugs. The next decade will determine how EBTs and incretin-based therapies can be optimally integrated into a combined, precision-based treatment algorithm for MASLD.

## Figures and Tables

**Table 1:** Endoscopic Bariatric and Metabolic Therapies: Mechanisms and Weight Loss Outcomes.

Procedure	Mechanism	Weight Loss Outcomes
Endoscopic Sleeve Gastroplasty (ESG)	Endoscopic suturing device plicates the greater curvature of the stomach, creating a tubular reduced-calorie reservoir without resection or incisions	15–20% TBWL over 12–18 months
Intragastric Balloon (IGB)	Saline- or gas-filled balloon occupies gastric lumen space, triggering stretch receptors and promoting early satiety	10–15% TBWL during 6-month indwelling period
Duodenal-Jejunal Bypass Liner (DJBL)	60 cm impermeable sleeve anchored in the duodenal bulb, bypassing nutrient exposure in proximal small intestine and mimicking Roux-en-Y bypass physiology	~12% TBWL, 20–25% excess body weight reduction at 12 months
Duodenal Mucosal Resurfacing (DMR)	Hydrothermal ablation of duodenal mucosa disrupts maladaptive nutrient sensing and regenerates epithelium	~5% TBWL over 6–12 months
